# Preliminary LC-MS Based Screening for Inhibitors of *Plasmodium falciparum* Thioredoxin Reductase (*Pf*TrxR) among a Set of Antimalarials from the Malaria Box

**DOI:** 10.3390/molecules21040424

**Published:** 2016-03-28

**Authors:** Neil K. Tiwari, Priscilla J. Reynolds, Angela I. Calderón

**Affiliations:** Department of Drug Discovery and Development, Auburn University, 4306 Walker Building, Auburn, AL 36849, USA; nkt0001@auburn.edu (N.K.T.); jzy0028@tigermail.auburn.edu (P.J.R.)

**Keywords:** *Plasmodium falciparum* thioredoxin reductase, antimalarial, screening for mechanism of action, Malaria Box

## Abstract

*Plasmodium falciparum* thioredoxin reductase (*Pf*TrxR) has been identified as a potential drug target to combat growing antimalarial drug resistance. Medicines for Malaria Venture (MMV) has pre-screened and identified a set of 400 antimalarial compounds called the Malaria Box. From those, we have evaluated their mechanisms of action through inhibition of *Pf*TrxR and found new active chemical scaffolds. Five compounds with significant *Pf*TrxR inhibitory activity, with IC_50_ values ranging from 0.9–7.5 µM against the target enzyme, were found out of the Malaria Box.

## 1. Introduction

Malaria continues to be a serious global health concern both due to the limited access for people in endemic regions to existing drugs and the increasing resistance of the parasite *Plasmodium falciparum*. The World Health Organization (WHO) reported 584,000 deaths in 2014 alone [1]. Research into novel antimalarial therapeutics will improve control and cost-effectiveness of interventions to eliminate the disease [2].

*Plasmodium falciparum* thioredoxin reductase (*Pf*TrxR) is a relatively novel drug target for the design of new antimalarials. Because infection with *P. falciparum* leads to increased oxidative stress in red blood cells, the parasite must have an efficient antioxidant removal system to prevent damage caused by reactive oxygen species. The effect is primarily achieved by a functional low molecular weight thiol-thioredoxin system utilizing *Pf*TrxR [3,4]. Because *P. falciparum* infected erythrocytes are susceptible to oxidative stress, activity of the enzyme has been shown to be essential for an adequate intracellular redox environment during intraerythrocytic development and proliferation, and consequently is necessary for survival of the parasite [5]. However, the IC_50_ values for *Pf*TrxR inhibition are at least 10-fold higher than the IC_50_ values for growth, indicating that *Pf*TrxR is likely to be a secondary target in killing the parasite.

Current therapeutics recommended by WHO are artemisinin-based combination therapies (ACTs) which utilize artemisinin in conjunction with alternative antimalarials, such as mefloquine and sulfadoxine/pyrimethamine. The logic behind these drugs is to hinder or prevent antimalarial resistance of artemisinin by pairing it with another drug. These cocktails have drawbacks in high cost, adverse drug reaction, and kinetic disparities between components which may even lead to increasing drug resistance to the ACT as a whole [6].

In order to catalyze the development of new antimalarials, MMV and the pharmaceutical company SCYNEXIS assembled the Malaria Box, an accessible collection of 400 non-toxic natural and synthetic chemotypes identified by phenotypic screening for asexual intraerythrocytic stages of *Plasmodium falciparum* [7]. Half of these compounds were selected based on their drug-like properties and the others as molecular probes. The mechanisms of action and the activity of these compounds in other stages of the parasite’s life cycle remain to be determined which make the set of compounds worth exploring for drug discovery.

The goal of this project was to identify novel chemical scaffolds with mechanisms of action through inhibition of *Pf*TrxR in the malaria parasite from the Malaria Box. Promising molecules with new mechanisms of action will catalyze drug development and are essential in combating growing antimalarial resistance. 

## 2. Results and Discussion

**Figure 1:**
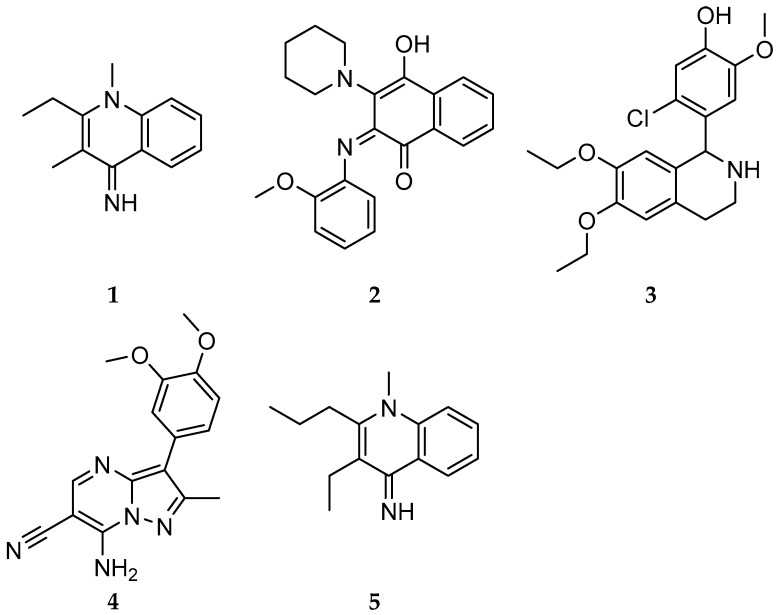
Structures of *Pf*TrxR inhibitors (**1**–**5**).

Two hundred and fifty compounds were screened against *Pf*TrxR at 10 μM due to their inclusion in the Malaria Box, indicating their established antimalarial activity and chemical diversity. By using a pre-established functional assay [8], five compounds (Figure 1 and Table 1) showed *Pf*TrxR inhibition greater than 50% at 10 μM and were selected for IC_50_ value determination. *Pf*TrxR inhibitory activity of the compounds are displayed in Table 2 alongside auxiliary antimalarial data provided by MMV: The EC_50_ values against *Pf*3D7, a chloroquine sensitive and sulfadoxine resistant strain of *Plasmodium falciparum*, and the IC_50_ against the parasite in MRC-5 cells.

**Figure 2:**
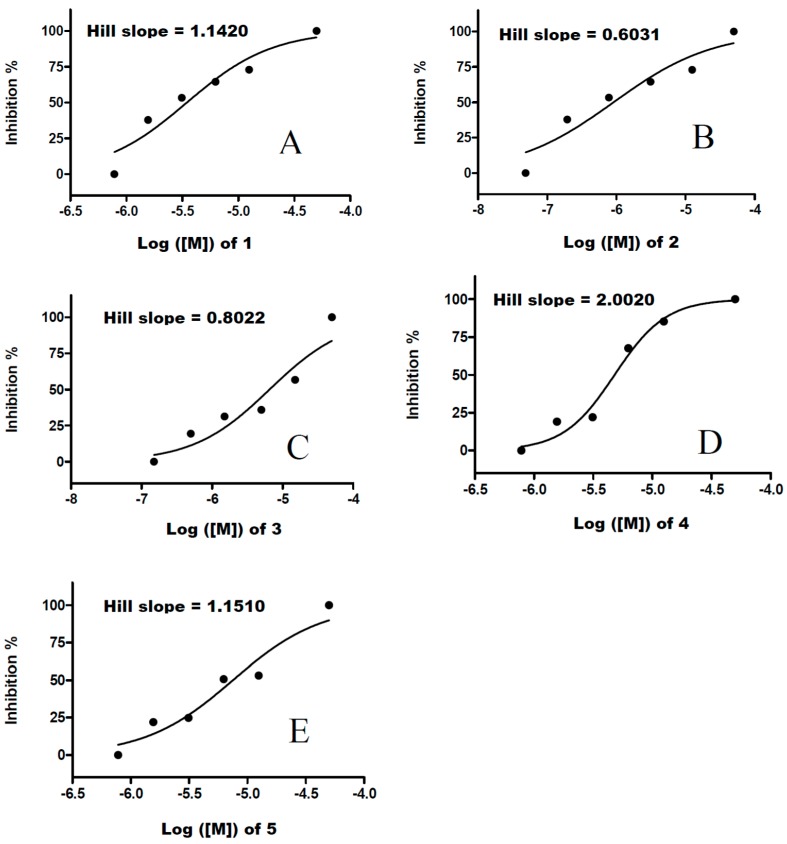
Dose response curves of compounds **1** (**A**); **2** (**B**); **3** (**C**); **4** (**D**) and **5** (**E**) tested against *Pf*TrxR.

Compound **2** was the best inhibitor of the target and most active against *Pf*3D7. It showed an IC_50_ of 0.9 μM against the target enzyme compared to the 3.5–7.5 μM of the other compounds tested (Table 2). The shallowness of the dose-response curve [11] and the lowest Hill slope of compound **2** (Figure 2) in comparison to the other four compounds may suggest non-specific binding to *Pf*TrxR.

The antimalarial studies against the *Pf*3D7 strain corroborated our findings, with compound **2** being by far the most active against this chloroquine sensitive strain. The antimalarial activity of the compound even surpassed traditional drugs such as artemisinin and chloroquine. Further experiments on compound **2** require verifying specificity and the antimalarial mechanism of action through testing oxidative stress in parasitized red blood cells in order use it as a prototype for chemical optimization of novel antimalarials. 

On the other hand, compound **4** displayed a steeper dose-response curve based on the high Hill slope (Figure 2) even though it showed the highest IC_50_ value against *Pf*TrxR and weak antimalarial activity. The other compounds (**1**, **3**, **4** and **5**) also inhibited the target, but their antimalarial activity was weaker than drugs currently available on the market. The fact that the IC_50_ values for *Pf*TrxR inhibition of the five compounds are at least 10-fold higher than the EC_50_ values for *P. falciparum* growth inhibition, indicates that *Pf*TrxR is likely to be a secondary target in killing the parasite. 

Another specific inhibitor of *Pf*TrxR has been identified recently as 6,7-dinitroquinoxaline. It demonstrates a mechanism of uncompetitive inhibition of *Pf*TrxR as a nitrophenyl derivative. 6,7-Dinitroquinoxaline is also proved being active in the lower micromolar range on the chloroquine-resistant *P. falciparum* strain *in vitro* [12]. Other inhibitors of *Pf*TrxR namely, 1,4-napthoquinone, 4-nitrobenzothiadiazole and menadione with antimalarial activity have been described by Munigunti *et al.* [8]. Finally, seven *Pf*TrxR inhibitors containing electrophilic moieties were identified from a subset of antimalarial compounds tested from the Tres Cantos antimalarial compound set (TCAMS) [13]. None of the *Pf*TrxR inhibitors identified by the listed research team share similar structural features with compounds (**1**–**5**).

Bis-(2,4-dinitrophenyl)sulfide (2,4-DNPS), a standard inhibitor for the *Pf*TrxR enzyme, was also evaluated against *Pf*TrxR as a positive control. Previous studies by our group reported moderate antimalarial activity of 2,4-DNPS against chloroquine-sensitive (D6) strains of *P. falciparum* IC_50_ 91.2 ± 11.3 µM, and chloroquine-resistant (W2) strains of *P. falciparum* IC_50_ 72.3 ± 11.3 µM [8]. 

Atovaquone, a known antimalarial drug, is inactive against *Pf*TrxR by displaying IC_50_ values higher than 10 µM. Atovaquone is used as a negative control in the *Pf*TrxR inhibitory assay. Cisplatin, a known anticancer agent, was used as a positive control in MRC-5 cell assay.

Compounds **1** and **5** have similar structures against MRC-5 cells through its extra bulk, differing only by one extra carbon in each side chains. The IC_50_ value of compound **1**, however, is nearly double that of compound **5** indicating that the extra carbons increase cytotoxicity. These compounds were only tested against cytosolic *Pf*TrxR and were not tested against human Thioredoxin reductase in this project. Cytosolic *Pf*TrxR is the *Pf*TrxR 1 isoform.

## 3. Experimental Section 

### 3.1. Reagents

Solvents used for LC-MS analysis were purchased from Fischer Scientific International (Atlanta, GA, USA). Test compounds were provided by Medicines for Malaria Venture (MMV) (Geneva, Switzerland). Deionized water generated by a Milli-Q water system (Millipore, MA, USA) was used in the experiments. *Pf*TrxR (M_r_ 59 kDa) enzyme was provided as a gift by Prof. Katja Becker, Justus-Liebig University, Giessen, Germany. The recombinant *Pf*TrxR was prepared and purified using silver-stained SDS page according to the procedure published by Kanzok *et al.* [14]. The specific activity of *Pf*TrxR (1.9 U/mg) was determined by DTNB [5,5’-dithiobis (2-nitrobenzoic acid)]. Protein concentration of enzymes was determined by Bradford method [15].

### 3.2. Test Compounds

The Malaria Box itself is an open access library [16] composed of 400 compounds originally identified by phenotypic screening of over 4,000,000 compounds from the research libraries of Saint Jude Children’s Research Hospital, Novartis, and GlaxoSmithKline [17]. It is a diverse set of 200 drug-like compounds as starting points for oral drug discovery and development and 200 probe-like compounds for used as biological tools in malaria research from the Malaria Box assembled and supplied by MMV. The thresholds for the antimalarial and cytotoxic activities of the 250 Malaria Box for this study were EC_50_: <500 µM and IC_50_: >50 µM, respectively. These pure synthetic compounds have been screened *in vitro* against of *P. falciparum* 3D7, a chloroquine sensitive but sulfadoxine resistant strain, for antimalarial activity and MRC-5 cells (human fetal lung cells) for cytotoxicity. 

### 3.3. LC/MS Based PfTrxR Inhibitory Activity Assay

The test compounds (10 µM in 1% dimethyl sulfoxide [DMSO]) were incubated with 0.5 µM *Pf*TrxR enzyme and 200 mM NADPH in the assay buffer (PE buffer: 100 mM potassium phosphate and 2 mM EDTA, pH 7.4). The enzymatic reaction was initiated by addition of 2.5 mM thioredoxin disulfide (Trx–S_2_) and incubated at 25 °C for 30 min. After the incubation, the reaction was quenched by addition of 0.1% formic acid. The reaction mixture was filtered through a 30 kDa MWCO ultrafiltration membrane centrifuged at 13,000 rpm at 20 °C for 10 min. The enzyme was trapped on the membrane and product thioredoxin dithiol (Trx–(SH)_2_) formed in the reaction was recovered in the filtrate and analyzed by LC-MS. A control experiment (without inhibitor) was performed under the same conditions described earlier. A calibration curve using known amounts of Trx–(SH)_2_ was prepared to quantify the amount of Trx–(SH)_2_ produced in the reactions [18]. 

An Agilent 1220 rapid resolution liquid chromatography (RRLC) system (Agilent Technologies, Santa Clara, CA, USA) coupled to the mass spectrometer was used to develop an assay to identify the *Pf*TrxR inhibitors. The MS settings used were as follows: nebulizer drying gas at 10 L/min, nebulizer pressure at 30 psig, gas temperature at 350 °C, capillary voltage at 4000 V, fragmentor at 250 V, skimmer at 60 V. The acquisition rate was at 1 scan/s scanning from 300–3200 *m*/*z*. The mass accuracy is 1/100 of a Dalton. 

### 3.4. Data Analysis

Chromatogram data from the LC/MS was extracted by Agilent Masshunter Software which is used to deconvolute multiply charged protein ions to the molecular weight that distinguishes Trx–S2 from Trx–(SH)_2_ easily due to a mass accuracy of 1/100 of a Dalton. The major mass/charge values of the multiply charged protein envelope in the Trx–(SH)_2_ spectrum 973.727, 1062.4212, and 1460.4540 were deconvoluted to proteins at the mass (*m/z* 11675.5410) of Trx–(SH)_2_ and used for relative quantitation by displaying the extracted ion chromatogram. The peak intensity of the control compound is compared to the test compound to quantify inhibition. By comparing the peak areas of Trx–(SH)_2_ in the control compound and the tested compounds, relative peak area ratios are calculated. These ratios are used to determine the inhibitory activity of the ligands on *Pf*TrxR.

For determination of IC_50_ values, the concentration of compound was titrated from 50 µM down to 0.07 µM incrementally by a factor of ⅓. Active compounds were compared against artemisinin and chloroquine, known antimalarial drugs. Additional data regarding the EC_50_ against *Pf*3D7, a chloroquine sensitive but sulfadoxine resistant strain of *Pf* TrxR and the IC_50_ against MRC-5 cells was also presented.

## 4. Conclusions 

The most promising molecule in this preliminary screening, compound **2**, showed an IC_50_ of 0.9 µM against *Pf*TrxR. Further experiments on this compound include verifying its mechanism of action through testing increased oxidative stress in parasitized red blood cells in order to consider this compound as a potential lead through the inhibition of *Pf*TrxR. A correlation of the extent of reported antimalarial and *Pf*TrxR inhibitory activities for the five most active compounds suggest that this mechanism of action may be secondary.

## Figures and Tables

**Table 1:** Identification of the five active *Pf*TrxR inhibitors.

Compound Number	MMV	IUPAC Name
**1**	MMV006278	2-ethyl-1,3-dimethylquinolin-4-imine;hydroiodide
**2**	MMV085203	2-(2-methoxyanilino)-3-piperidin-1-ylnaphthalene-1,4-dione
**3**	MMV008956	5-chloro-4-(6,7-diethoxy-1,2,3,4-tetrahydroisoquinolin-1-yl)-2-methoxyphenol
**4**	MMV396797	7-amino-3-(3,4-dimethoxyphenyl)-2-methylpyrazolo[1,5-*a*]pyrimidine-6-carbonitrile
**5**	MMV008416	3-ethyl-1-methyl-2-propylquinolin-4-imine;hydron;iodide

**Table 2:** *Pf*TrxR inhibitory, antimalarial and cytotoxic activities of the most active Malaria Box compounds.

Test Compounds	*Pf*TrxR IC_50_ (µM)	*Pf*(3D7) ^a^ EC_50_ (nM)	MRC-5 Cells ^b^ IC_50_ (µM)
**Compound 1**	3.5	378	41.5
**Compound 2**	0.9	5.3	68.7
**Compound 3**	6.6	370	85.4
**Compound 4**	4.8	477	>100
**Compound 5**	7.5	242.5	71.2
**2,4 DNPS**	0.5	D6/W2 [8]	ND
**Atovaquone**	>10	0.24 [9]	ND
**Cisplatin**	ND	ND	19.6
**Artemisinin**	ND	20.7	ND
**Chloroquine**	ND	27.3	ND

*Pf*3D7: Chloroquine sensitive but sulfadoxine resistant strain of *P. falciparum*; MRC-5 cells: Human fetal lung cells; ND: Not determined. ^a,b^ EC_50_ and IC_50_ values obtained from MMV database [10].
